# Public Finance, Fiscal Rules and Public–Private Partnerships: Lessons for Post-COVID-19 Investment Plans

**DOI:** 10.1057/s41294-023-00213-x

**Published:** 2023-02-27

**Authors:** Alessandra Cepparulo, Giuseppe Eusepi, Luisa Giuriato

**Affiliations:** Sapienza University of Rome, via Castro Laurenziano 9, 00161 Rome, Italy

**Keywords:** Public–private partnerships, Fiscal illusion, Budget constraints, Fiscal rules, Investment, European union, C23, H54, L32, E62

## Abstract

We explore the distribution of public–private partnerships (PPPs) among the European Union countries, with a special focus on fiscal rules and budgetary constraints while controlling for empirically identified drivers. While offering the opportunity to increase innovation and efficiency in the public sector infrastructure, PPPs allow governments to relax their budget and borrowing constraints. We find that the state of public finances influences the government’s choice of PPPs and makes them more appealing for reasons other than efficiency. Stringent numerical rules on the budget balance also foster government’s opportunism in the choice of PPPs. On the other hand, high levels of public debt increase the country risk, and discourage private investors from PPP contracts. The results highlight the importance of restoring PPP investment choices based on efficiency criteria and adapt fiscal rules to shield public investment while stabilizing private expectations by means of credible trajectories of debt reduction. The findings contribute to the debate on the role of fiscal rules in fiscal policy and of PPPs in infrastructure financing.

## Introduction

The COVID pandemic, as well as the demographic, technological and climate challenges that all countries are facing, require new strategies of public action and larger ambitions on public investments. In many European Union (EU) countries, this implies a reversal of a long trend of investment decline that has reduced public capital stock and the availability of infrastructure. The lack of investment means that the quality of capital stock suffers from deficiencies, deterioration, and poor conditions, even where its quantity of remains high, while new needs are emerging and will challenge governments’ capacity to devise solutions to them.

In many EU countries, the new investment strategies will be undertaken in the context of high debt, raising concerns about its sustainability, and reduced fiscal space in public budgets. The next return to fiscal rules and adjustments is also expected and governments will have to articulate it while preserving their investment policies and creating incentives for the private sector’s participation: “public finance needs to lead the way, private actors need to provide the scale” (European Commission 2020).

In the past years, EU countries’ investment has been compressed by the lack of fiscal space and budgetary consolidation operations that have impacted disproportionately on capital expenditures, because policymakers refrain from cutting government consumption to avoid voters’ frustration and find it easier to resort to investment cuts (Balassone and Franco 2000; Blanchard and Giavazzi 2004; Mehrotra and Välilä 2006). Furthermore, when fiscal policy is constrained by domestic and supranational fiscal rules, it can increase its tendency to pro-cyclicality and dump the adjustment on investment (Easterly 1999; Galì and Perotti 2003; Breunig and Busemeyer 2012), although evidence is debated (Bergman and Hutchison 2015; Gootjes and de Haan 2020; Keita and Turcu 2022) and the design of rules seems to be effective in mitigating the negative impact on the composition of public expenditures (Ardanaz et al. 2021; Guerguil et al. 2017).

The need to build public infrastructure, while facing financial restrains and abiding by budgetary rules, has increased governments’ interest in public–private partnerships (PPPs) and will make them relevant in the new investment policies. PPPs are long-term cooperative risk-sharing agreements between a public entity and a private partner for financing, building, and operating a public infrastructure together with a significant component of private finance. Return on private capital is generated according to different arrangements. In government-funded PPPs the government provides predetermined payments (per volume of services provided or per number of users) for making the asset available or ensuring the supply of services. In user-funded PPPs, the private provider recoups its infrastructure investment through charges to end users. Up-front capital subsidies to the initial investment can be included in the arrangement, as well as public guarantees on risks and compensation clauses in the event of the early rescission of the contract.

Although the effect of PPPs on the government’s intertemporal balance sheet is like that of traditional public provision (Engel et al. 2014) and the cost of financing is usually higher than the cost of public funds (Blanc‐Brude and Strange, 2007), PPPs are attractive to national and local authorities since they allow for the delivery of infrastructure while deferring its payments to the future-also to future administrations-and promise innovation and increased efficiency in the public sector thanks to the involvement of private businesses. The promises of these long-term contracts are delivered if the design and the key conditions are met, such as pricing arrangements that correctly reflect the risks transferred to each party, realistic forecasts of demand risks, contract incentives and penalties for infrastructure/service quality, and prior comparative analyses of different procurement options.

In presence of public spending constraints imposed by market access conditions or by fiscal rules, PPPs provide the opportunity for governments to relax their budget and borrowing constraints in the short term and allow policymakers more discretion to fund investment (Mühlenkamp 2014; Engel et al. 2020). However, whenever risks are not adequately transferred to the private partners or the government allows remuneration rates on private capital that do not correspond to the risks borne or too generous direct or indirect guarantees, PPP projects “become quasi-public, but with the funding removed from the government’s balance sheet” (Benito et al. 2008, 965). This creates the fiscal illusion (Puviani 1903) that partnerships are much less expensive than traditional public procurement. Besides, when accounting rules allow for off-balance sheet registration of PPPs, debt and deficit hiding motivations add and create an unwarranted bias in favor of the partnerships (Välilä, 2005; Tanzi 2015).

These considerations invite to a closer examination of PPPs and their interplay with budgetary constraints and fiscal rules. The choice of PPPs has already been examined by the literature in relation to the state of public finances (Antellini Russo and Zampino, 2012; Buso et al. 2017; Albalate et al. 2015; Mazzola et al. 2019; Mota and Moreira 2015), but, to the best of our knowledge, not in relation to various types of domestic and supra-national rules and their stringency.

Fiscal rules are generally introduced to address the challenge of containing or reducing rising deficits and debts while increasing fiscal sustainability. They also seek to minimize negative externalities-like in the European Monetary Union-, and to limit policymakers’ discretion, thereby increasing policy credibility, but they also be a source of negative unintended side effects. In particular, numerical fiscal rules foster the government’s budgetary opportunism to circumvent them and encourage the use of fiscal gimmicks, creative accounting practices (Milesi-Ferretti 2004; von Hagen and Wolff 2006) and implicit liabilities, which conceal the long-run impact of fiscal measures on debt and future expenditures (Easterly 1999; Milesi-Ferretti and Moriyama 2006). Too rigid fiscal rules frameworks and the need to achieve strict budgetary discipline can lead to the search of new financing tools to develop investment projects while hiding their debt and deficit impact. However, the fiscal illusion motive can vanish or be downscaled when flexibility clauses or the explicit consideration or protection of investment are included.

We aim at assessing if different types of rules are associated to the use of PPPs. Budget balance, expenditure and debt rules impact differently on the possibility of funding investments and hence on the opportunity to resort to PPPs. The next revision of the Stability and Growth Pact and the numerous proposals advanced have increased the interest in the variety of rules, which include multi-year ceilings on primary expenditures (Giavazzi et al. 2021), golden rules for the digital and climate transition (Darvas and Wolff 2021; Bordignon and Pisauro 2021), expenditure ceilings consistent with debt targets (Caselli et al. 2022), medium-term debt ceilings (Martin et al. 2021). We are also interested in the relevance of rules vis à vis the state of public finance, specifically whether financial restraints imposed by budgetary conditions prime over the restrictions imposed by fiscal rules in driving opportunistic strategies tied to PPPs. We analyze PPPs, budgetary constraints and different types of numerical fiscal rules in the EU countries, drawing our data from the IMF Investment and Capital Stock Dataset (2021 release, IMF 2021a) and estimating a fixed-effects regression with Driscoll and Kraay (1998) standard errors over the years 1990–2019.

PPPs account for a small share of EU countries’ public investment, but they are a complementary source of investment in politically appealing sectors that allow for the direct reward of private investors (energy, telecommunications, infrastructure, transports). After the financial crisis, the EU PPP market did not completely collapse, but it now moves at a much slower pace: transactions reaching a financial close fell from about € 30 bn. in the record year 2007 to € 8 bn. in 2021 (EIB 2022).

**Fig. 1:**
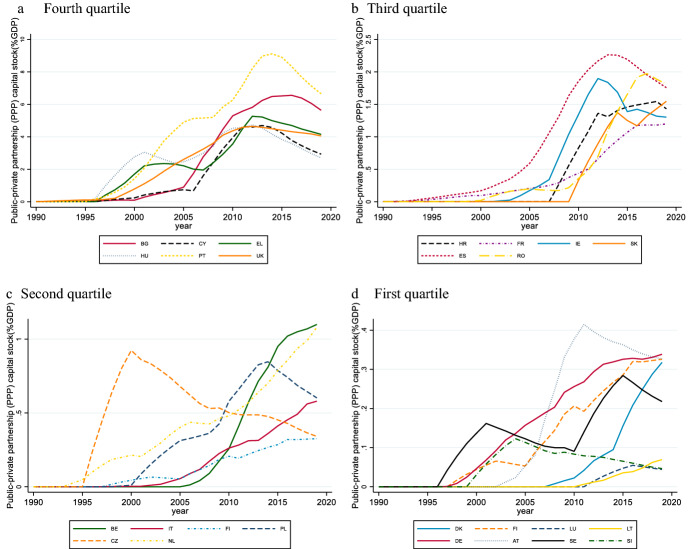
Countries distribution according to PPP volumes (1990-2019; % GDP).* Source*: own elaboration on IMF (2021a)

Even though the EU directives have established a very favorable and uniform legislation for PPPs and imposed binding norms aimed at ensuring fair competition, the uptake of PPPs across the EU countries has been very uneven (Fig. 1). In terms of PPP capital stock, the United Kingdom, Greece, Portugal, and Bulgaria are the most active markets. Spain and Portugal accounted for large shares of the overall PPP market value in the mid-1990s and 2000s, thanks also to very large projects. Instead, Northern countries and many Eastern European countries have shown less appetite for partnerships.

The paper is organized as follows. "Literature Review" Section presents the literature review. In "Methodological approach" Section, we present the database, the variables employed, and the research method and "Results and discussion" section discusses the main results. “Conclusions” Section concludes.

## Literature Review

The large amount of capital required by infrastructure puts a strong pressure on national budgets and it is hardly affordable when public decision-makers face binding fiscal and financial constraints. By limiting the upfront costs, deferring and spreading public sector payments through time-especially when the full accounting of financial constraints can be avoided-PPPs offer a possible way out, but they also create an “affordability illusion, […] the illusion that a PPP project can take place because the financing is there, but forgetting that the project eventually has to be paid for and the financing paid back” (Yescombe and Farquharson 2018, p. 100). Unless adequately reported in the balance sheet, PPPs foster voters’ fiscal illusion by obscuring the level of spending and the tax requirements associated with it (McQuaid 2019; Boardman et al. 2016).

Indeed, public finance factors and governments’ strategies of avoiding excessive public borrowing are found as significant determinants of PPPs (Benito et al. 2008; McQuaid and Scherrer 2010; Vecchi et al. 2010; Russo and Zampino, 2012; Cruz and Marques 2011; Fernandes et al. 2015; Reeves 2015; Bergere 2016; Albalate et al. 2015; van den Hurk 2018; Jensen and Dowlatabadi 2018). Van den Hurk et al. (2016) argue that budgetary reasons were essential for the choice of PPPs in Southern EU member states, while Petersen (2010) finds that, where the public finance constraints were less compelling, countries (e.g., Denmark and Sweden) have been less eager to opt for PPPs.

A large literature (Benito et al. 2008; Acerete et al. 2019; Reeves 2015; Cepparulo et al. 2019 among others) documents the explicit use of PPPs to circumvent budgetary restrictions thanks to the off-balance sheet accounting of the partnership transactions. In this respect, the EU accounting rules have contributed to the preference for PPPs, because, under certain conditions-amounting to enough risks being transferred to the private partners-, the contracts do not show on the government balance sheet, and the share of PPP-related debt is not considered for compliance with the Stability and Growth Pact (SGP)^1^ (Benito et al. 2008; Cruz and Marques 2011; Reeves 2015; Bergere 2016; Engel et al. 2020). The focus on risk sharing rather than on the budgetary impact of PPPs has been maintained by Eurostat in its subsequent revisions of the accounting rules. In general, Eurostat allows the off-balance sheet registration of user-funded PPPs and, under some conditions (e.g., the absence of government guarantees), also that of government-funded PPPs.

The choice of PPPs to circumvent budgetary constraints may expose the public sector to higher-than-expected costs, and to running into contingent liabilities related to the presence of guarantees that may be triggered by a future event and that are difficult to evaluate in amount and timing. These guarantees transfer the financial risk to taxpayers and, when called, they may cause large sudden outlays for the public sector (as was the case of Greek motorways in 2013 and 2015). Thus, PPPs off-balance accounting treatment implies an underestimation of the future burden on taxpayers (Stafford et al. 2010; Fernandes et al. 2015). Bova et al. (2016) estimate the average fiscal cost of PPP contingent liabilities realization at about 1.2% GDP^2^.

Fiscal rules have been tested for their impact on public investment but not on PPPs. However, the design and stringency of rules are relevant for governments’ investment strategies, including those that have an opportunistic component. Budget balance rules are associated with sub-optimal levels of investment (Dur et al. 1999; Krogstrup and Wyplosz 2009; Afonso and Jalles 2015; Ardanaz et al. 2021). Golden rules permit borrowing only to finance investments, thus protecting them during cyclical downturns and budgetary consolidations (Guerguil et al. 2017), but they can also open to opportunistic definitions of capital expenditures and disincentivise adequate cost-benefit analysis (Balassone and Franco 2000; Servén, 2007). Expenditure rules set ceilings that usually include at least part of the capital spending and that risk distorting the expenditure composition unless flexibility mechanisms to counter the cycle or to insulate items of infrastructure investment are provided (Cordes et al. 2015; Ljungman 2009; Marinheiro 2021). Rules expressed in terms of structural balance or providing for escape clauses grant some protection to investment along the economic cycle (Ardanaz et al. 2021). Therefore, rules embedding margins for manoeuvre for investment (item exclusion, temporary deviations from targets, room under expenditure ceilings, flexibility between budget years) should reduce the need for alternative methods of investments, including PPPs.

The considerations of public finance combine with the politicians’ incentive to opt for PPPs to increase their political consensus in times of curtailed budgets (Cappellaro and Longo 2011; Reeves 2015) and in the run-up to elections. In many instances, political convenience has led governments to choose PPPs, claiming that there was no other viable alternative, and dismissing the need to show their value for money (Hall 2008). The political convenience of PPPs is multi-dimensional (Coghill and Woodward 2005), because partnerships not only help politicians to please the electorate by providing services and avoiding upfront costs, but they also free revenues to be used for other targets and help public decision-makers gain the recognition of good management. The presence of significant political benefits to the political party or ruling interests in power contributes to explain governments’ preference for PPPs (Hodge and Greve 2009; Boardman et al. 2016).

The political strategies related to PPPs are, however, constrained by the presence of institutional factors. Institutional actors who control the public investment process can stabilize the environment for PPP decision-making (Savitch 1998). Bertelli et al. (2020) argue that the political risk to which PPPs are exposed can be reduced by increasing political stability–i.e., the number of veto points that make the political environment more predictable. This keeps politicians from intervening in the project, increase private firms’ confidence and reduces the probability of PPP cancellation. Reyes-Tagle and Garbacik (2016) conclude that proper institutional controls and safeguards are necessary to avoid accruing unsustainable fiscal liabilities in countries using PPPs for budgetary reasons. Controlling from this conceptual framework, we analyze the role played by the presence and strength of fiscal rules and budgetary conditions and test their impact on the adoption of PPPs.

## Methodological Approach

Our dependent variable^3^ ($ppp$) is the PPPs investment flow (in billions of constant 2017 international dollars, purchasing power parity adjusted) from the IMF database^4^. It is characterized by a high incidence of zeros (around 40% of the sample) that correspond to real observations. Previous literature coped with this issue by estimating^5^ either a Tobit model (Mazzola et al. 2019; Checherita 2009; Banerjee, et al. 2006) as a corner solution model^6^, or a panel data model (Random effect: Kasri and Wibowo 2015; Mengistu 2013, Fixed effect: Moszoro et al. 2017; Panel Corrected Standard Errors: Mota and Moreira 2015), or a two-stage model like the Heckman election model (Reyes-Tagle and Garbacik 2016).

Preliminary analysis of the data guides our model selection. According to the Hausman test^7^ (given the presence of heteroscedasticity checked via the Wald test^8^), the fixed-effects estimator is better fitted for our sample. As standard error estimates are severely biased if not appropriately accounted for, we verify the presence/absence of cross-section independence^9^ and contemporaneous correlation^10^ (Wooldridge test). The result of the tests points to the existence of serial correlation while cross-sectional dependence is absent. Therefore, we prefer to use an estimator which contemporarily addresses both departures from the canonical assumptions: heteroscedasticity and autocorrelation. Then, we estimate a fixed-effect model with clustered standard errors (eq.1) for the EU countries observed over 30 years (1990-2019):

1 $${\mathrm{ppp}}_{\mathrm{it}}={\beta }_{0}+{\beta }_{1}{\mathrm{econ}}_{\mathrm{it}-1}+{\beta }_{2}{\mathrm{fiscal}}_{\mathrm{it}-1}+{{\beta }_{3}{\mathrm{pubfin}}_{\mathrm{it}}+\beta }_{4}{\mathrm{instit}}_{\mathrm{it}}+ {\varepsilon }_{\mathrm{it}}$$

where, the subscript *i* denotes the country and the subscript *t* denotes the year. The disturbance term is given by two error components,${\varepsilon }_{\mathrm{it}}={\alpha }_{i}+{{\delta }_{t}+ u}_{\mathrm{it}}$ with ${\alpha }_{i}$ representing the country effect, which we assume to be a fixed effect, including cultural and historic aspects, by assumption correlated with the regressors. The term ${u}_{it}$ is the stochastic error, while ${\delta }_{t}$ represents time effect^11^.

As a robustness check and in order to address the zeros issue, we also estimate a Poisson fixed-effect model (Table 5, Appendix). According to Wooldridge (1999) this estimator produces meaningful results even when the dependent variable is not a count variable and it applies to any situation with nonnegative outcomes, including zeros. In addition any variance-mean relationship and any serial correlation are allowed.

Real GDP and financial variables are expressed in logarithm and lagged. In this way the regressors are predetermined with respect to the dependent variable and reverse causality should be excluded. No collinearity issues pertain to the model. The VIF is below 2 for all the regressors 12.

First, we investigate the role of fiscal rules. During the examined period, fiscal rules were modified to deal with the global financial crisis, their number was increased and, in general, they were changed into so-called “second generation” rules that are meant to be more flexible and enforceable with improved mechanism for monitoring (Eyraud et al. 2018). As already observed, the empirical literature supports the idea that stricter fiscal rules promote better fiscal performances, but also increase the risk that governments behave opportunistically and shift from overt to hidden forms of borrowing. As fiscal rules reduce the options available to governments, especially in times of fiscal consolidation when the burden of adjustment is placed on investments, PPPs can provide an attractive alternative to finance infrastructure. Conversely, when fiscal rules are less strict or allow for some flexibility or protection in the treatment of capital expenditure, the incentive to resort to PPPs should be weaker.

EU countries adopt both domestic and EU supranational rules and have different procedures, institutions, and agencies to apply and monitor them. Although the EU rules have undergone several modifications^13^, from the original Maastricht Treaty targets on deficit and debt, in none of its versions the SGP provides for investment exclusion. However, since 2011, it allows for an investment allowance in the preventive arm by relaxing the medium-term objective convergence obligation during depressed economic conditions. Domestic numerical fiscal rules include limits or ceilings on the budget balance, debt, expenditures and revenues.

To summarize the presence and features of fiscal rules, we build an index of national fiscal rules stringency (*nat_rules*) and an index of overall stringency, combining supranational and national fiscal rules (*overall_rule)*, based on IMF Fiscal Rules Dataset (Table 1). Following Schaechter et al. (2012), this index is obtained by summing up the sub-indices by type of rules. On its turn, each sub-index is built by translating the institutional characteristics (sectoral coverage, monitoring procedures, enforcement procedures, legal basis and institutional supporting procedures) of the rule into a score and summing them up into a single measure (normalized between 0 and 1).

**Table 1:** Definition of variables and expected result

Variable	Description	Source	Expected coefficient
**Fiscal Rules**	Fiscal rule indices (normalized):	IMF	
*nat_rule*	National fiscal rules index	IMF	+
*overall_rule*	Overall index of fiscal rules	IMF	+
*exp_rule*	Expenditure rule index	IMF	+/–
*bb_rule*	Budget balance rule index	IMF	+
*debt_rule*	Debt rule index	IMF	+/–
**Public finance**			
*bbalance*	General Government net lending (+)/ net borrowing (–)	CPDS	–
*debt*	General Government gross debt	CPDS	–
*totrev*	Tax revenues	CPDS	+/–
**Economy**			
*Gdp*	Real GDP	IMF	+
*int_rate*	Real long-term interest rate	CPDS	–
*exp*	No of years of experience with PPPs	IMF/EIB	+
*capital*	General government capital stock	IMF	–
**Politics /institutions**			
*checks*	Number of veto players	QoG	–
*right/left/centre*	Left/right centre-wing governments	CPDS	+/–
*fraction*	Political fragmentation	QoG	–

*CPDS*: Comparative political dataset; *QoG*: Quality of government dataset

We also consider the normalized indices of three different kinds of domestic fiscal rules that constrain the budget balance (*bb_rule)*, public debt (*debt_rule)*, and public expenditures (*exp_rule*) respectively^14^. In 2020, most of the considered countries adopt a domestic budget balance rule, 11 out of 25 countries also have a debt rule and 14 have an expenditure rule.

The stringency of the rules depends on their legal basis^15^, coverage, monitoring of compliance, presence of escape clauses and independent control bodies. Under the hypothesis that more stringent rules negatively affect investment and increase the preference for PPPs, we expect a positive coefficient for the *nat_rules* and *overall_rule* variables. Besides, in line with the evidence on rules and investments, we expect a positive coefficient also for the budget balance rule. Instead, the coefficient of expenditure rules is unpredictable as these rules are articulated in different ways and can allow for flexibility over the cycle or some shield of investment. Debt rules also have an unpredictable effect, as they display many different features-e.g., political commitments to achieve a reduction in the central government debt-GDP ratio (e.g., Finland, Bulgaria), debt targets with the protection of infrastructure projects (Luxembourg), debt-interest-to-revenue rules (United Kingdom).

We group the other control variables into subsets of regressors (Table 1). Public finance variables help us assess the importance of the fiscal constraints. In presence of strained public finances and reduced available resources, the short-term opportunity to record infrastructure assets out of the government’s balance sheet (Auriol and Picard 2013, p.191) increases the favor for PPPs. We employ the General Government net lending/borrowing (*bbalance,* negative value for deficit, positive value for surplus*)* and debt (*debt*) and test the hypothesis that sounder public finances make the choice of PPP less necessary or attractive. Hence, we expect a negative sign for the budget balance, which is also the variable where the surveillance under the SGP has been stricter, and the incentive for fiscal illusion higher.

Similarly to the budget balance, the debt variable could indicate a condition of financial restraints and hence increase the appeal of PPPs. However, high levels of public debt also increase the private sector’s perception of the country risk and undermine the investors’ confidence, including their interest in PPPs. Indeed, the attractiveness of PPPs depends on the expectation of returns that not only outperform market averages but are also comparatively safe. High public debts represent a special concern for private investors, as the measurement of debt is more robust and less biased than that of the deficit (Kezbere and Maurer 2018). We support this latter hypothesis and expect a negative sign for the debt coefficient.

To complete the picture of public finance conditions, we consider tax revenues expressed as the ratio of total tax revenues to GDP (*totrev*): the sign of the coefficient is, in principle, ambiguous. On the one hand, high taxes correspond to high levels of recurring expenditures, leaving little room for discretionary spending (Reyes-Tagle and Garbacik 2016) and fostering infrastructure investments via PPPs (Checherita 2009). On the other hand, the availability of large tax revenues should reduce the need of alternative sources of investment financing (Rosell and Saz-Carranza 2019; Albalate et al. 2015).

The third subset of variables includes country-level controls to account for the domestic economic structure and the business environment. Many potential consumers and bigger markets–proxied by the real GDP (*realgdp)*-are an incentive for private partners’ participation in PPPs. The amount of capital stock (*capital)* is also considered because it should decrease the need to finance new infrastructure investment via PPPs. Besides, given that considerable administrative skills are necessary for the design and implementation of PPP contracts, previous experience represents both a reputational capital (Galilea and Medda 2010) and a catalyst of future success (Ng et al 2012). We consider the number of years with positive investment in PPPs as representative of the level of expertize (*exp*) and expect a positive sign, given that countries are more likely to implement PPPs investment the more experienced they are with such programs. Finally, we employ the lending interest rate (*int_rate*) to proxy the discount rate used in PPPs investment decision.

Finally, a fourth subset of variables is employed to test the relevance of political and institutional factors that may discipline the political potential of PPPs. We test for the role of checks and balances, which increase political stability, reduce the political risk faced by businesses, and influence public managers’ investment decisions. We expect that their presence reduces the opportunistic employment of PPPs, as it impacts on the fiscal illusion logic by limiting the possibility for politicians to employ PPPs for pork-barrel projects at the benefit of their constituencies (Maskin and Tirole 2008). The variable employed is *checks* from the Quality of Government (QoG) database^16^.

Government’s political orientation is captured by the relative power position (*right/left/centre*) of the parties in government as measured by their share of seats in the parliament. According to Savitch’s (1998) analysis for the United Kingdom, the propensity for PPPs seems to be associated to left parties supporting a larger provision of public services. However, Li (2003) finds that PPPs have become the trend in the country, independently of the party to power. Albalate et al. (2015) confirm the pragmatic—rather than the ideological—origin of the choice of PPPs in the US. Given the not conclusive evidence, we do not form any prior on the expected sign of the coefficients.

To analytically account for political competition, we employ the government’s fractionalization index (*fraction*) from the QoG database^17^. The more fragmented the government, the more heterogeneous the preferences of politicians and their attempts to satisfy conflicting demands by means of private sector's resources. Accordingly, we expect a negative coefficient for the variable. Table 4 (Appendix) provides the statistical summary of the employed variables.

## Results and Discussion

We first present the results for the indices of national and overall fiscal rules strength (Table 2). In all specifications, the index of national fiscal rules shows the expected sign, but it is not significant. Similar result is found when considering the overall index, combining supranational and national fiscal rules. This means that, in general, the presence of stronger fiscal rules does not correlate with a preference for PPP investment.

**Table 2:** Fixed effect model with clustered standard errors: national and overall fiscal rules indices

	National fiscal rules strength			Overall fiscal rule strength		
*GDP*	0.88**	0.92**	0.91**	0.65***	0.61***	0.67***
	(−2.27)	(−2.38)	(−2.25)	(−3.42)	(−3.02)	(−3.1)
*exp*	0.01*	0.01*	0.01*	0.01***	0.01**	0.02***
	(−2.04)	(−1.89)	(−2.03)	(−3.06)	(−2.71)	(−3.26)
*interest*	−0.007	−0.006	−0.009	−0.02***	−0.02***	−0.0219**
	(−0.57)	(−0.52)	(−0.83)	(−2.87)	(−3.03)	(−2.49)
*debt*	−0.08*	−0.10**	−0.07	−0.05	−0.07	−0.0425
	(−2.05)	(−2.33)	(−1.54)	(−1.27)	(−1.62)	(−0.86)
*bbalance*	−0.51**	−0.58**	−0.60**	−0.43	−0.453	−0.553
	(−2.07)	(−2.51)	(−2.19)	(−1.30)	(−1.40)	(−1.39)
*totrev*	0.61**	0.76***	0.54*	0.51	0.595	0.532
	(−2.27)	(−2.91)	(−1.86)	(−1.49)	(−1.7)	(−1.44)
*capital*	−0.26	−0.27	−0.29	−0.12	−0.119	−0.131
	(−1.57)	(−1.61)	(−1.64)	(−1.06)	(−1.03)	(−1.05)
*checks*	−0.02	−0.02*	−0.02	−0.02**	−0.0254**	−0.017
	(−1.63)	(−1.75)	(−1.15)	(−2.14)	(−2.53)	(−1.52)
*fraction*	0.21*	0.18	0.14	0.22**	0.192*	0.182*
	−1.74	−1.59	−1.14	(−2.48)	(−2.04)	(−1.78)
*left*	0.0009***			0.001***		
	(−3.34)			(−4.04)		
*right*		−0.001**			−0.001***	
		(−2.74)			(−3.82)	
*centre*			−0.0004*			−0.0004
			(−1.73)			(−1.47)
*nat_rule*	0.10	0.14	0.09			
	(−0.83)	(−1.16)	(−0.68)			
*overall_ rule*				−0.01	0.04	−0.03
				(−0.07)	−0.35	(−0.25)
*_cons*	−3.05	−3.35	−2.34	−2.43	−2.32	−2.03
	(−1.38)	(−1.53)	(−1.09)	(−1.05)	(−0.98)	(−0.79)
*N*	404	404	404	544	544	544
*R-sq*	0.35	0.35	0.31	0.36	0.36	0.31

^*^p<0.05, **p<0.01, ***p<0.001

However, when we consider different kind of rules (Table 3), we observe that more stringent rules on the budget balance are positively and significantly related to an increased use of partnerships. This complements the already observed distortionary effects of budget balance rules on public investment.

**Table 3:** Fixed effect model with clustered standard errors: individual fiscal rules

	Expenditure rule			Budget balance rule			Debt rule		
*GDP*	1.16*	1.10*	1.20*	0.84**	0.88**	0.90**	0.74*	0.79*	0.73*
	(−1.84)	(−1.77)	(−1.83)	(−2.18)	(−2.31)	(−2.23)	(−1.89)	(−2.03)	(−1.83)
*exp*	0.009	0.009	0.009	0.01**	0.01**	0.01**	0.01**	0.01**	0.01**
	(−1.6)	(−1.54)	(−1.55)	(−2.16)	(−2.1)	(−2.19)	(−2.19)	(−2.1)	(−2.18)
*interest*	−0.01**	−0.01***	−0.01***	−0.001	−0.0002	−0.001	−0.001	−7.00E−05	−0.003
	(−2.77)	(−2.92)	(−3.49)	(−0.06)	(−0.01)	(−0.09)	(−0.07)	(−0.00)	(−0.21)
*debt*	0.04	0.037	0.06	−0.08*	−0.09**	−0.07	−0.07	−0.07	−0.06
	−0.62	−0.62	−0.89	(−1.83)	(−2.07)	(−1.48)	(−1.63)	(−1.70)	(−1.33)
*bbalance*	−0.35	−0.51	−0.25	−0.46*	−0.51**	−0.55*	−0.03	−0.16	−0.05
	(−0.75)	(−1.01)	(−0.47)	(−1.78)	(−2.12)	(−1.93)	(−0.09)	(−0.50)	(−0.14)
*totrev*	0.63*	0.59*	0.57*	0.59**	0.72***	0.53*	0.62**	0.70**	0.59**
	(−2.09)	(−2.02)	(−1.88)	(−2.37)	(−2.97)	(−2.03)	(−2.39)	(−2.69)	(−2.23)
*capital*	−0.59*	−0.6	−0.61	−0.27*	−0.28*	−0.31*	−0.17	−0.19	−0.18
	(−1.75)	(−1.73)	(−1.70)	(−1.72)	(−1.76)	(−1.88)	(−1.13)	(−1.20)	(−1.15)
*checks*	−0.02	−0.02	−0.01	−0.02*	−0.03**	−0.02	−0.02	−0.02	−0.01
	(−1.07)	(−1.13)	(−0.66)	(−1.98)	(−2.14)	(−1.60)	(−1.58)	(−1.66)	(−1.12)
*fraction*	0.16	0.13	0.12	0.19	0.18	0.13	0.24*	0.22*	0.18
	(−1.32)	(−1.15)	(−1.18)	(−1.54)	(−1.44)	(−1.04)	(−1.96)	(−1.85)	(−1.51)
*left*	0.0007*			0.0008***			0.0009***		
	(−1.83)			(−3.09)			(−3.24)		
*right*		−0.0007			−0.0009**			−0.001**	
		(−1.50)			(−2.53)			(−2.68)	
*centre*			−0.0007*			−0.0003			−0.0005***
			(−1.97)			(−1.35)			(−2.95)
*exp_rule*	0.007	0.01	0.003						
	(−0.11)	(−0.16)	(−0.04)						
*bb__rule*				0.11	0.14*	0.14			
				(−1.44)	(−1.74)	(−1.62)			
*debt_rule*							−0.14	−0.12	−0.18
							(−1.50)	(−1.21)	(−1.63)
*_cons*	−4.09	−2.76	−4.47	−3.04	−3.37	−2.55	−5.01**	−4.86**	−4.68*
	(−1.35)	(−0.98)	(−1.34)	(−1.29)	(−1.44)	(−1.12)	(−2.08)	(−2.10)	(−1.94)
*N*	254	254	254	398	398	398	392	392	392
*R-sq*	0.42	0.41	0.40	0.35	0.34	0.31	0.34	0.33	0.30

^*^p<0.05, **p<0.01, ***p<0.001

As Afonso and Jalles (2015) show for the EU countries using indices constructed by the European Commission, stronger budget balance rules decrease the amount of resources for public investment: we complement their findings by showing how these rules make PPPs an attractive alternative to increase policy discretion and finance infrastructure in countries under pressure to limit their budget deficits. This confirms that stringent budget balance rules are prone to incentivise policy choices to circumvent the rule itself and a distorted employment of PPPs with implications on their design and risk sharing arrangements.

The lack of significance of expenditure rules coefficients may be related to the fact that, although investment is not completely excluded from the expenditure ceilings, it is usually treated separately from other expenditure categories (Ljungman 2009) or allowed extra-ceiling financing (e.g., from financial disinvestment as in Finland, or from special funds as in the Netherlands), while margins under the ceiling absorbe cyclical fluctuations and shield investment. Debt rules are also less constraining than budget balance rules as they are usually not clearly operational (Schaechter et al. 2012): their impact on investment (as confirmed by Guerguil et al. 2017) and PPPs is thus limited.

The results in Tables 2 and 3 show that countries are influenced in their PPP choice more by the state of their public finance and the ensuing availability of resources than by fiscal rules. A worsening in the government’s budgetary position is significantly associated with a higher use of PPPs in many specifications. This result is in line with the econometric results of Albalate et al. (2015) for the US states, Buso et al. (2017) and Antellini Russo and Zampino (2012) for French and Italian municipalities respectively, and studies based on surveys and case studies (Benito et al. 2008; McQuaid and Scherrer 2010; Vecchi et al. 2010; Cruz and Marques 2011; Fernandes et al. 2015; Reeves 2015; Bergere 2016; Albalate et al. 2015; van den Hurk 2018). The positive and significant coefficient of tax revenues suggests that governments finance infrastructure via PPPs when the taxpayers’ burden is high (as in Checherita 2009). This confirms that PPPs offer policymakers a viable alternative to build and operate public infrastructure without further increases in taxes while pleasing their electorate and fostering fiscal illusion thanks to the deferral of payments.

The hypothesis on public debt is confirmed by the negative and significant coefficient, pointing to the relevance of sustainability concerns. High levels of public debt increase the country risk, reduce the government’s credibility, and discourage private investors. This result complements the empirical evidence in Bacchiocchi et al. (2011) who point to high levels of public debt distorting the allocation of public expenditure and hampering public investment. We observe a difference between results for central and sub-national governments. Russo and Zampino (2012) find a strong positive relationship between PPPs and local public debt, and Albalate et al. (2015) also find a similar result for the US states. Sub-national governments are, however, more constrained in their access to the financial markets and, hence, more inclined to opt for PPPs, while the central government enjoys larger self-finance capacity and market access. This latter is, however, limited by credibility and risk sustainability factors that may drive investors away.

As for the economic variables, GDP is always a significant determinant, while the signs of the other variables are as expected, but not always significant. The hypothesis for the positive role played by the past experience with PPPs (*exp*) is strongly confirmed as in Mota and Moreira (2015). Indeed, public agencies need time and skills to build the necessary institutional arrangements and the capacity to handle PPPs projects-for example through the creation of PPPs units or agencies, standardized contracts or procedures for evaluation and implementation (Hodge et al. 2018; Verhoest et al. 2015).

When turning to the political and institutional variables, we find that political fragmentation and its conflicting demands increase the use of PPPs. In terms of political orientation, left-wing governments are more supportive of partnerships (as in Mota and Moreira 2015), while centre and right-wing governments display negative and significant coefficients^18^. The negative sign of the coefficient for the number of checks and balances confirms–as in Bertelli et al. (2020)-the expectation of a positive role of institutional veto points to control the investment decisions of public sector officials, reduce the ‘political’ risk and the risk of accruing unsustainable fiscal liabilities. The result, however, is not always significant.

These results point to the need of budgetary provisions that shield public investment also in times of economic stress and when public finances are overstretched to avoid that PPP are chosen to overcome financial constraints. Simple budget balance rules are not suited to this purpose and should be adapted to protect investment by means of golden rules or their variants^19^, clear escape clauses that allow for temporary deviations from the targets (Ardanaz et al. 2021; Guerguil et al. 2017), provisions to isolate investment from the cycle, or special public infrastructure agencies (Mintz and Smart 2006).

Besides, to bring the choice of PPPs back to efficiency criteria, while avoiding distortions in the investment decision and the distribution of risks in the project, accounting rules on off-balance-sheet registration should be revised. Transparency principles should be applied to record PPP-related contingent liabilities, which expose governments to the risk of severe fiscal problems. On-balance-sheet recording, at least in internal documentation as in France, could be a first important step for all EU countries. Other complementary provisions to mitigate the fiscal illusion bias could be employed, such as spending caps to public officials (Maskin and Tirole 2008), ceilings for the stock of PPP-related contingent liabilities, maximum annual payment amounts for PPPs, or independent agencies giving advice on PPP contracts and performance.

Robustness checks are displayed in Table 5 (Appendix). We estimate a Poisson fixed effect model on the same sub-samples, where the coefficient of the budget balance rule turns non-significant. The public finance variables are, instead, highly significant, confirming that the budget and debt conditions are important drivers of the distribution of PPPs. Economic, institutional and political factors are also confirmed to influence the relationship between PPPs and fiscal rules and budgetary conditions.

## Conclusions

Public finances in the EU countries are strained by the COVID-19 pandemic and the war in Ukraine while public debts and deficits have reached unprecedented levels. At the same time, in the coming years governments will need to provide the infrastructure required by the digital and climate transitions and recover their backlog of investments. These policy choices will deeply impact on the future development of European countries and the dynamics of their public finances, which will be constrained also by restored or revised fiscal rules. All this makes PPPs attractive for public authorities in search of alternatives to increase investment resources without further worsening their budgetary position.

We focus on the role of fiscal rules and budgetary constraints in influencing the appeal of PPPs in the EU countries. Evidence of the impact of fiscal rules on investment is still scarce, while no study has been performed on their relationship with PPPs. We observe that, in their choice of PPPs, countries are constrained more by the state of their public finances and the fiscal space available than by their fiscal rules. More than any other type of rule, strong budget balance rules are positively associated with larger employment of PPPs to allow infrastructure financing.

The state of the budget balance reduces the fiscal space available for infrastructure financing and calls for the involvement of the private sector in funding and managing infrastructure, also thanks to the possibility of off-balance sheet accounting treatment. Off-balance sheet registration of PPP projects calls for attention because, if PPPs are employed to avoid financial constraints in the short term, in the medium/long term they create unsustainable fiscal liabilities, unless adequate controls and safeguards are implemented. On the other hand, high levels of public debt increase the country risk perception and reduce the attractiveness of PPPs for the private partners. Therefore, only credible trajectories of debt reduction will be able to stabilize private expectations.

This complex picture calls for revising fiscal and accounting rules to reduce the scope of fiscal illusion and the incentives to circumvent the rules themselves. At the same time, the necessary fiscal adjustments and debt reduction policies that many countries will need in the next future should not undermine the space for public investment so that PPPs can still provide an attractive alternative to finance infrastructure, but an efficiency-based one.

## Data Availability

All authors contributed to the study conception, design, data collection, analysis and the preparation of the final manuscript.

## Appendix

See Tables

**Table 4:** Descriptive statistics

Variable	Obs	Mean	Std. Dev.	Min	Max
gdp	765	5.7	1.3	2.3	8.4
exp	800	7.2	7.7	0.0	30.0
int_rate	645	5.5	3.7	-0.2	24.1
debt	725	4.0	0.7	-1.0	5.7
bbalance	702	4.6	0.0	4.2	4.7
totrev	726	3.8	0.2	3.2	4.1
capital	711	5.2	2.0	-1.3	10.0
checks	772	4.2	1.4	1.0	16.0
fraction	779	0.4	0.3	0.0	0.8
nat_rules	442	0.4	0.2	0.0	0.8
left	766	39.3	37.3	0.0	100.0
right	766	39.4	37.8	0.0	100.0
centre	766	19.4	27.9	0.0	100.0
nat_rules	273	0.4	0.2	0.0	1.0
bb_rules	436	0.4	0.3	0.0	1.0
debt_rules	430	0.3	0.3	0.0	1.0
overall_rules	587	0.5	0.2	0.0	0.8

^4^ and

**Table 5:** Robustness checks: Poisson fixed-effects model

	Budget balance rule		
*GDP*	2.85^**^	2.98^**^	3.74^***^
	(−2.16)	(−2.06)	(−2.63)
*exp*	0.09^***^	0.08^**^	0.07^*^
	(−2.59)	(−2.33)	(−1.8)
*interest*	0.06	0.06	0.05
	(−0.78)	(−0.83)	(−0.76)
*debt*	−0.9^***^	−1.13^***^	−0.97^***^
	(−5.04)	(−5.28)	(−3.48)
*bbalance*	−2.948^***^	−3.214^***^	−3.234^***^
	(−3.64)	(−4.25)	(−3.07)
*totrev*	2.73^**^	3.15^***^	2.01^*^
	(−2.36)	(−2.73)	(−1.94)
*capital*	−1.79^***^	−1.93^***^	−2.14^***^
	(−3.53)	(−3.69)	(−4.04)
*checks*	−0.20^***^	−0.19^***^	−0.15^**^
	(−3.29)	(−3.24)	(−2.04)
*fraction*	1.22^***^	0.99^**^	0.85
	(−2.65)	(−2.05)	(−1.51)
*left*	0.007^***^		
	(−5.55)		
*right*		−0.005^***^	
		(−3.68)	
*cent*			−0.006^**^
			(−2.44)
*bb_rule*	−0.058	0.068	0.1
	(−0.11)	(−0.13)	(−0.17)
*N*	393	393	393

^*^*p*<0.05, ***p*<0.01, ****p*<0.001

Public finance, fiscal rules and public–private partnerships: lessons for post-COVID-19 investment plans

5.

## Footnotes

1 PPP-related assets are classified off the balance sheet of the government if: (1) the private partner bears the construction risk (e.g., late delivery or additional costs), (2) the private partner bears either availability (volume and quality of output) or demand risk (variability of demand), and (3) the risks are not incurred by the government through other means (e.g., governmental guarantees or early redemption clauses).

2 Fiscal illusion impacts also on the characteristics of the PPP deals (Fernandes et al. 2015; van den Hurk 2018), determines distortions, delays, and cancellation of some public investment decisions (Reeves 2015). It may prime over the project’s merit or its value for money (Acerete et al. 2019) and is responsible for low-quality expensive projects (Engel et al. 2014) or for pushing public authorities in sectors where PPPs do not add value (Riess 2005).

3 We don’t take the PPPs variable log-linearized as in presence of heteroschedasticity the estimates are inconsistent (Santos Silva and Tenreyro 2006).

4 The IMF database includes total PPP projects commitments taken from the European Investment Bank (EIB) for European countries and from the World Bank Private Participation in Infrastructure database for low- and middle-income countries. EIB data are sourced from its European PPP Expertize Center (EPEC) (see IMF 2021b). They record PPPs at the time of financial close and include the total value of PPP projects above 5 million Euros. The included PPP projects are long-time investment projects planned by central governments. See Kappeler and Nemoz (2010) for methodological issues.

5 On the contrary when the dependent variable was represented by the number of projects the most used approach was the Random-Poisson model (Kasri and Wibowo 2015; Hammami et al. 2006; Sharma 2012; Enders 2008).

6 “Interestingly, Tobin’s original application to spending on consumer durables is clearly a corner solution application, and he never uses the word ‘‘censored’’ in his article” (Wooldridge 2010, chapter 17, p. 670).

7 Hausman test to choose between a fixed effects model or a random effects model: chi2(11) = 40.61, Prob>chi2 = 0.0000.

8 Wald test for heteroschedasticity: chi2 (25) = 18502.77, Prob>chi2 = 0.0000.

9 Friedman's test of cross-sectional independence = 9.720, Pr = 0.9956.

10 Woolridge test for autocorrelation: F(1, 24) = 95.187, Prob > F = 0.0000.

11 Test for time dummies results: F( 24, 24) = 5.11, Prob > F = 0.0001

12 Previous studies consider the population size as a further regressor in order to catch the demand-driven request for public services as well as the need of gaining new competences from the private sector. When introducing this variable together with the real GDP variable (measuring the market size) the variance inflation factor of population and GDP variable becomes higher than 10 highlighting multicollinearity issues. For this reason, we consider only the real GDP as a measure of the market.

13 Until the Great Recession, the SGP was focused on deficit targets and the corrective arm, but its implementation was less than satisfactory. The preventive arm, fiscal surveillance in the European semester, an expenditure rule and a debt rule were operationalized only after the 2011 reform.

14 We don’t consider golden rules given the insufficient number of countries adopting it.

15 The legal basis is mostly statutory, but four countries introduce the budget balance rule at the constitutional level.

16 The indicator is incremented by one for each of the following cases: if there is a chief executive, if the chief executive is competitively elected and if the opposition controls the legislature. In parliamentary systems, the checks index is incremented by one for every party in the government coalition provided that it is necessary to maintain a majority, and for every party in the government coalition that has a position on economic issues closer to that of the largest opposition party rather than to that of the party of the incumbent executive.

17 It measures the probability that two randomly chosen deputies from the government parties belong to different parties.

18 This result is in contrast with evidence for French municipal governments in Buso et al. (2017), where PPPs are mostly chosen by municipalities in the right-wing party rather than by those in the centre party.

19 For example, Mintz and Smart (2006) suggest the introduction of a limit to debt financing of the capital budget expressed in terms of GDP or a fixed debt-to-GDP ratio for public capital.
